# Meandering pulmonary veins: Two case reports

**DOI:** 10.1097/MD.0000000000019815

**Published:** 2020-04-17

**Authors:** Miji Lee, Kyung Nyeo Jeon, Mi Jung Park, Kyungsoo Bae

**Affiliations:** a Department of Radiology, Institute of Health Sciences, Gyeongsang National University School of Medicine, Jinju; b Department of Radiology, Gyeongsang National University Changwon Hospital, Changwon, Republic of Korea.

**Keywords:** arteriovenous malformation, meandering pulmonary vein, pathogenesis, scimitar syndrome, scimitar variant

## Abstract

**Rationale:**

Meandering pulmonary vein is a rare congenital pulmonary vascular anomaly. It presents unilateral single pulmonary vein that takes a circuitous route in the lung and drains normally into the left atrium. Most cases of meandering pulmonary vein have been reported to be right-sided. A few of them coincided with features of scimitar syndrome.

**Patient concerns:**

A 71-year-old woman and a 20-year-old man presented with incidentally found abnormal findings on chest radiographs.

**Diagnosis:**

Through multi-detector chest computed tomography, the 71-year-old woman was diagnosed as left-sided meandering pulmonary vein without any other anomalies while the 20-year-old man was diagnosed as having right-sided meandering pulmonary vein with features of scimitar syndrome.

**Interventions:**

Specific intervention was not performed for either patient.

**Outcomes:**

These patients were reassured and discharged. They are doing well without any respiratory symptoms.

**Lessons:**

Meandering pulmonary veins can occur on the left side and coincide with features of scimitar syndrome. Multi-detector computed tomography with 3D reconstruction allows clear depiction of vascular connections and associated anomalies, obviating the need for invasive procedures.

## 1 Introduction

Meandering pulmonary vein (MPV) is a rare anomaly of pulmonary vein. It is often confused with more common conditions such as scimitar syndrome and pulmonary arteriovenous malformation (AVM). Scimitar syndrome is a complex malformation characterized by 3 main abnormalities, including the right pulmonary vein draining into IVC, a systemic arterial supply of the lung, and hypoplasia of the right lung.^[1–3]^ Other associated anomalies include cardiac malformations and bronchial anomalies.^[4]^ We report 2 rare cases of MPV. The first one was a left sided MPV. The second one was a right sided MPV with some features of scimitar syndrome. We report image findings of these 2 cases of MPV, with a review on the embryological basis of pulmonary venous anomalies to help physicians understand its pathophysiology and decide management plans.

## 2 Case report

This study was approved by the Institutional Review Board of Gyeongsang National University Changwon Hospital. Each patient provided written consent for publication of the case.

### 2.1 Case 1

**Figure 1:**
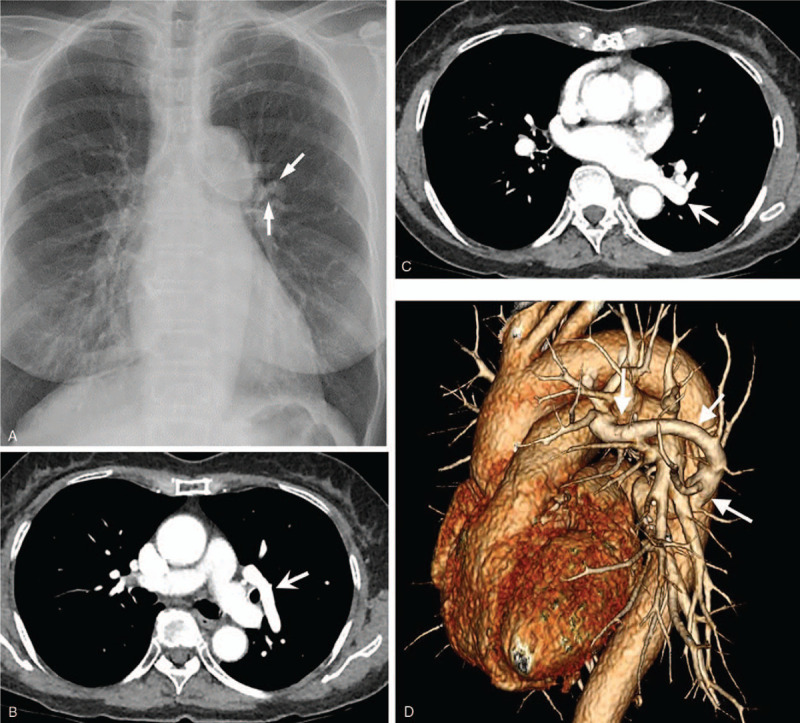
A 71-year-old woman presented with abnormal finding on chest radiograph during medical check-up. A. Chest radiograph showing a small dense curved opacity (arrows) in the left perihilar area. B and C. Chest CT images revealing an unusual vascular anomaly draining the left upper lobe (arrow in B) and joining the left inferior pulmonary vein (arrow in C) before merging into the left atrium. D. 3D volume rendered image showing an enlarged aberrant anomalous vein (arrows) courses tortuously to posterior direction and drains into the left atrium.

A 71-year-old woman was transferred to our hospital for further evaluation of abnormal chest radiographic finding on medical check-up. She was previously healthy without any specific medical history. The patient did not complain any respiratory symptoms. Chest radiograph showed a dense curved opacity in the left hilar area, suspicious of vascular malformation (Fig. 1A). Contrast-enhanced chest computed tomography (CT) scan was taken using a 256 multi-detector scanner. Chest CT revealed unusual vascular anomaly in the left lung. The pulmonary vein draining the left upper lobe passed tortuously to the posterior direction to cross the oblique fissure and join the left inferior pulmonary vein before merging into the left atrium (Figs. 1B and C). Three-dimensional (3D) volume rendered image comprehensively displayed anomalous course of the left pulmonary vein (Fig. 1D). There was no left to right shunt or other cardiovascular anomalies. The patient was diagnosed as having an incidental rare vascular variant of meandering left pulmonary vein. Because there was no hemodynamic disturbance, no intervention was performed. She was reassured and recommended to have annual follow-ups.

### 2.2 Case 2

**Figure 2:**
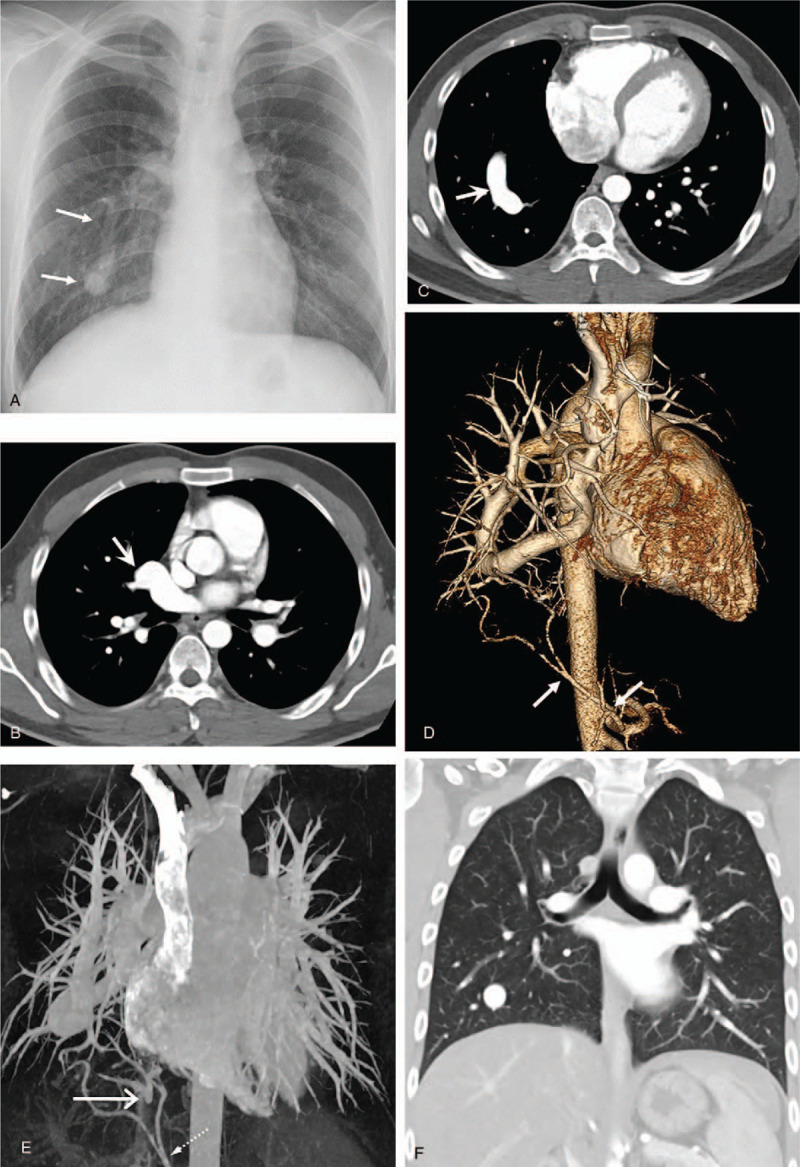
A 20-year-old man transferred for further evaluation of abnormal finding on chest radiograph. A. Chest radiograph demonstrating an elongated curvilinear structure (arrows) coursing vertically down the right heart border. B and C. Contrast enhanced chest CT images revealing an engorged anomalous pulmonary vein (arrow in B) emptying into the left atrium via left superior pulmonary venous ostium (arrow in C). There was no right inferior pulmonary vein (not shown here). D. Three-dimensional volume rendered image showing focal systemic arterial blood supply from the abdominal aorta in the posterior basal segment of the right lower lobe (arrows). E. Maximum intensity projection image demonstrating small pulmonary venous branches emptying into IVC (long arrow). Also note systemic arterial supply from abdominal aorta (dashed arrow). F. Coronal reformatted image with lung window setting showing 2 bilobed lungs with bilateral hyparterial bronchial branching pattern (left bronchial isomerism).

A 20-year-old man was transferred to our hospital for evaluation of an abnormal finding on chest radiograph detected during health screening. The patient did not complain any respiratory symptoms. Chest radiograph showed an elongated curvilinear structure coursing vertically through the right lower lung which then became kinked above the right diaphragm (Fig. 2A). This finding was suspicious of scimitar vein. However, his right lung volume and cardiac position were normal. Contrast enhanced chest CT revealed an engorged anomalous pulmonary vein. Right inferior pulmonary vein was absent. Anomalous pulmonary vein coursed downward toward the diaphragm before turning upward to empty into the left atrium via the left superior pulmonary venous ostium (Figs. 2B and C). In the posterior basal segment of the right lower lobe, focal systemic arterial blood supply from the abdominal aorta was found (Fig. 2D). In the same lung region, small pulmonary venous branches that merged to one before emptying into the inferior vena cava (IVC) were also observed (Fig. 2E). Both lungs showed a bi-lobed pattern with left bronchial isomerism (Fig. 2F). Therefore, the patient was diagnosed as having meandering right pulmonary vein with features of scimitar syndrome at the same time, although the amount of shunt flow was hemodynamically insignificant. Examinations including echocardiography and laboratory studies did not reveal any other abnormalities. Because the patient was hemodynamically stable without any symptoms, he was discharged without any further intervention.

## 3 Discussion

MPV is a very rare pulmonary venous anomaly with less than 20 reported cases.^[5]^ This vascular malformation can occur on both sides. The majority of reported cases were right-sided. Only one recent paper reported a left-sided one in a young woman,^[6]^ similar to our first case.

MPV can often be confused with scimitar syndrome, particularly when it occurs on the right side because both conditions show a prominent vertically running vascular shadow on chest radiographs (scimitar sign). Scimitar syndrome consists of anomalous pulmonary venous drainage of the right lung to the IVC, resulting in left-to-right shunt, anomalous arterial supply of the right lower lobe, and hypoplasia of the right lung, with resultant dextroposition of the heart.^[4,5]^ In MPV, anomalous vein takes tortuous intrapulmonary course, although the vein eventually drains normally into the left atrium.^[7]^ While each has a clear definition, some previous literatures have reported coinciding cases of MPV and scimitar vein,^[1,3,5]^ like our second case. In the first case, the patient had an isolated anomaly of left sided single pulmonary vein, coursing circuitously within the lung but drained normally into LA (MPV). In the second case, the patient had both MVP and scimitar vein. The vein showing scimitar sign on chest X-ray had tortuous course but drained normally into LA, which was a MPV. In addition, a small part of the right lower lobe drained separately into the IVC (Scimitar vein). The region was supplied by a small systemic artery arising from the abdominal aorta. The patient did not have other features of scimitar syndrome except left bronchial isomerism.

Apart from classic scimitar syndrome, various terms such as scimitar variant, incomplete scimitar sign, and scimitar vein with dual pulmonary venous drainage have been used for cases that lack all typical features of the scimitar syndrome and sometimes manifest additional features.^[1,3–5]^ Classic scimitar syndrome, scimitar variant, and MPV can be considered as specta of pulmonary anomalies having a common embryological basis.

Normal development of the pulmonary venous system during fetal life involves the loss of primitive connections of the pulmonary venous plexus to the splanchnic plexus. At the same time, the pulmonary venous plexus can establish connections with the newly developed common pulmonary vein which drains into the left atrium. If communication with the common pulmonary vein and the left atrium is interrupted, primitive connections of the pulmonary and systemic vascular beds may persist, resulting in partial anomalous pulmonary venous drainage.^[8]^ Delayed union of the pulmonary venous plexus with the common pulmonary vein could result in pulmonary venous connections with both systemic circulation and the left atrium, which may explain the dual drainage of the meandering pulmonary vein,^[8–11]^ like our second case. Subsequent obliteration of pulmonary venous connections with the systemic circulation could result in MPV draining exclusively to the left atrium.

Differentiation between venous anomalies is required to decide whether treatment is necessary. Our first case was similar to pulmonary AVM in chest radiograph. Our second case was confused with classic scimitar syndrome at first. However, multi-detector CT with 3D reconstruction allowed clear depiction of vascular connections and associated anomalies. Thus, it is important to understand characteristics of each vascular abnormality. Diagnostic distinction among scimitar syndrome, pulmonary AVM, and MPV is summarized in Table 1. Isolated MPV does not require any intervention. Treatment for pulmonary AVM and scimitar syndrome can require treatment depend on the amount of shunt and associated anomalies.

**Table 1:**
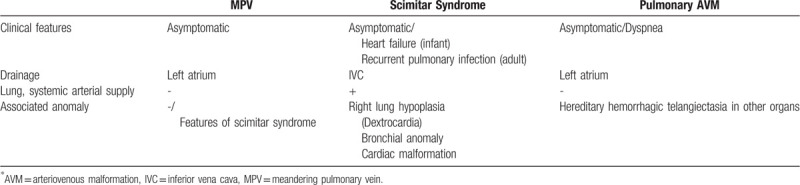
Diagnostic distinction among meandering pulmonary vein, scimitar syndrome, and pulmonary AVM.

In conclusion, we reported 2 rare cases of MPV, one occurring on the right side and one occurring on the left side. Multi-detector CT with 3D reconstructions enabled comprehensive understanding of vascular connections and associated anomalies, obviating the need for invasive procedures.

## Author contributions

**Conceptualization:** Kyungsoo Bae, Kyung Nyeo Jeon.

**Formal analysis:** Miji Lee.

**Investigation:** Miji Lee, Mi Jung Park.

**Methodology:** Miji Lee, Kyung Nyeo Jeon.

**Resources:** Kyung Nyeo Jeon, Mi Jung Park.

**Supervision:** Kyungsoo Bae, Kyung Nyeo Jeon.

**Writing – original draft:** Miji Lee, Kyung Nyeo Jeon.

**Writing – review & editing:** Miji Lee, Kyungsoo Bae, Mi Jung Park, and Kyung Nyeo Jeon.
